# Psychostimulants and social behaviors

**DOI:** 10.3389/fphar.2024.1364630

**Published:** 2024-04-25

**Authors:** Valeska Cid-Jofré, Tamara Bahamondes, Agustina Zúñiga Correa, Ivalú Ahumada Arias, Miguel Reyes-Parada, Georgina M. Renard

**Affiliations:** ^1^ Centro de Investigación Biomédica y Aplicada (CIBAP), Escuela de Medicina, Facultad de Ciencias Médicas, Universidad de Santiago de Chile (USACH), Santiago, Chile; ^2^ Facultad de Ciencias de la Salud, Universidad Autónoma de Chile, Talca, Chile

**Keywords:** psychostimulants, social behavior, reward, amphetamine, methamphetamine, cocaine, modafinil

## Abstract

Mounting evidence from animal models and human studies indicates that psychostimulants can significantly affect social behaviors. This is not surprising considering that the neural circuits underlying the regulation and expression of social behaviors are highly overlapped with those targeted by psychostimulants, which in most cases have strong rewarding and, consequently, addictive properties. In the present work, we provide an overview regarding the effects of illicit and prescription psychostimulants, such as cocaine, amphetamine-type stimulants, methylphenidate or modafinil, upon social behaviors such as social play, maternal behavior, aggression, pair bonding and social cognition and how psychostimulants in both animals and humans alter them. Finally, we discuss why these effects can vary depending on numerous variables such as the type of drug considered, acute *versus* long-term use, clinical *versus* recreational consumption, or the presence or absence of concomitant risk factors.

## 1 Introduction

The relationship between our species and drugs is ancient (de Wit and Richards, 2004) and the reasons for drug use are diverse: medical purposes, sociability, relaxation, focus and work, and awakening properties, among others. In the brain there are specific nuclei that organize, activate, and modulate the procurement of natural rewards such as food, social interactions, and sex. Some of these nuclei include the ventral tegmental area (VTA), nucleus accumbens (NAc), prefrontal cortex (PFC), lateral septum (LS) and ventral pallidum (VP), and they are part of the “reward circuitry” or mesocorticolimbic system (Hyman et al., 2006; Koob and Volkow, 2010). Noteworthy, these brain nuclei are not only “activated” by natural but also by other stimuli such as drugs of abuse (Volkow and Morales, 2015). This activation elicits a strong relationship between the effect of the stimulus (i.e., euphoria, pleasure, better attention, or cognitive performance) and the context where it was performed (Berridge and Kringelbach, 2015). Therefore, there exists a learning process leading the individual to repeat the behavior. It has been suggested that one of the main features of drug addiction (drug abuse) *versus* recreational use is the seeking of the reward without “liking” it (Berridge and Robinson, 2016), regardless of the adverse outcomes (an impulsive and compulsive intake).

Dopamine (DA) projections from VTA to NAc and other limbic structures participate in two crucial features of the rewarding process: motivated behavior and reinforcement of those behaviors. These characteristics are fundamental for drug addiction (Koob and Volkow, 2016; Volkow et al., 2016).

Psychostimulants like cocaine, amphetamine-type stimulants, methylphenidate (MPH), modafinil (MOD) and new psychoactive substances with stimulant effects are globally used. Estimates made by the United Nations in 2019 (Breckenridge et al., 2019), illustrate that there are 18 million cocaine users worldwide and confiscation of psychostimulants only in the Americas is close to 1,215 tons, being cocaine, methamphetamine (MAMPH) and amphetamine (AMPH) the leading drugs, although the percentage varies among continents.

Psychostimulants are drugs that act on the central nervous system (CNS), increasing alertness, and arousal and causing general behavioral excitement (McCreary et al., 2015). Their primary mechanism of action is to enhance the activity of the three main monoamine neurotransmitters: DA, norepinephrine (NE) and/or serotonin (5-HT), which produces an intense activation of several brain pathways underpinning the aforementioned behaviors (Sofuoglu and Sewell, 2009). Cocaine, AMPH, MPH, MOD, and MAMPH have the DA and NE transporter (DAT and NET, respectively) as main targets, and using differential mechanisms lead to an augmentation of the extracellular levels of DA and NE in the synaptic cleft (Goodwin et al., 2009; Underhill et al., 2020). On the other hand, drugs such as 3,4-methylenedioxymethamphetamine (MDMA or ecstasy), which is not necessarily considered a typical psychostimulant, increase mainly 5-HT levels acting on the corresponding transporter (Dunlap et al., 2018; Nichols, 2022). These diverse pharmacodynamics are likely related to some controversial effects that are discussed below. Since psychostimulants can generate various neurobiological effects, they have numerous clinical uses as well (Faraone, 2018). Psychostimulants are used as pharmacotherapy in neuropsychiatric disorders such as narcolepsy and attention-deficit/hyperactivity disorder (ADHD) (Faraone, 2018), but these drugs have been used for other purposes in non-medical settings and off-label fashion especially in young populations (Arria and DuPont, 2010).

Regarding ADHD, the psychostimulants with the highest number of prescriptions are AMPH and MPH. In addition, MOD is another stimulant that has shown promising results in clinical trials in children with ADHD (Rugino, 2007; Amiri et al., 2008; Goez et al., 2012; Arnold et al., 2014; Faraone, 2018; Caldwell et al., 2020; Zahed et al., 2022). Different studies have reported an over-prescription and illegal use of MOD, MPH and AMPH in young healthy individuals (Lakhan and Kirchgessner, 2012). However, it remains unknown the effects of the illicit use of these psychostimulants after long term (d’Angelo et al., 2017).

Considering our social nature, social interactions are regarded as natural rewards. Interactions with peers are essential for allowing allies to conform, protecting territories or relatives, pair bonding, parental and maternal care of offspring, shared foods, shelter, and more.

Social behaviors can be broadly defined as a modality of communication and/or interaction between two or more individuals of a given species of animals (Chen and Hong, 2018). Humans, as well as other species, depend on social stimuli to make decisions. Thus, for social behavior, individuals use highly complex communication methods, specie-specific sensory cues and dynamic information between conspecifics (Chen and Hong, 2018). These behaviors include a variety of interactions and settings like social group living, social organization, mating, parenting, aggression, social play, and others. It must be considered that different pharmacological interventions may affect social behavior performance and increase or decrease the interactions involved in them.

**FIGURE 1:**
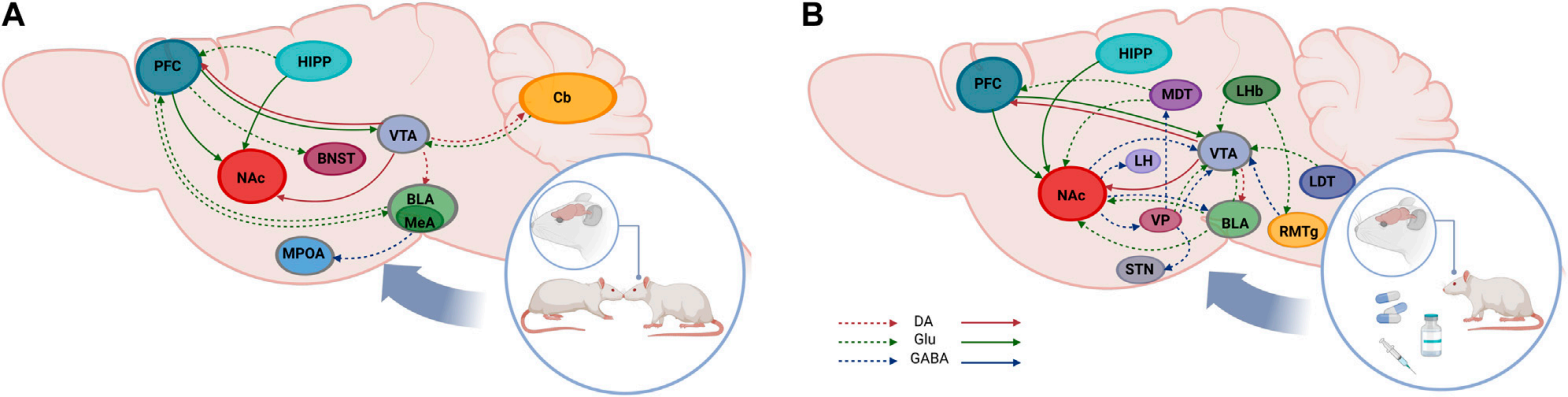
Schematic representation of brain pathways involved in social behavior and reward. Social behaviors are regulated by brain regions and connections that, to some extent, overlap to dose activated by drugs of abuse and natural reinforces (continuous arrows). Sagittal rodent brain section showing the different nuclei of the cortical mesolimbic system and their connections within the social neural circuit **(A)** and the reward neural circuit **(B)**. Ventral tegmental area (VTA), nucleus accumbens (NAc), prefrontal cortex (PFC), hi ppocampus (HIPP), bed nucleus of the stria terminalis (BNST), medial preoptic area (MPOA), cerebello (Cb), basolateral amygdala (BLA), medial amygdala (MeA), lateral hypothalamus (LH), ventral pallidum (VP), sub thalamic nucleus (STN), rostromedial tegmentum (RMTg) lateral habenula (LHb), laterodorsal tegmentum (LDT), dopamine (DA), glutamate (GLU).

Studies in rodents using the conditioning place preference (CPP) test have demonstrated the natural tendency to seek social interactions and their reward properties (Douglas et al., 2004; Thiel et al., 2008; Douglas et al., 2018), both in adults and juvenile individuals. Several brain areas have been associated with social behaviors such as PFC, infralimbic cortex (IL), NAc, VTA, hippocampus (Hipp), LS and others (Douglas et al., 2004; Solié et al., 2022; Yan and Rein, 2022). Interestingly, as we mentioned, some of them are also part of the reward circuitry. Therefore, social interactions are regulated by some of the same brain regions activated by drugs of abuse (Figure 1). Given the crucial role of social interaction in normal socio- and neuro-development (O’Connell and Hofmann, 2011; Gunaydin et al., 2014), it is fundamental to unravel how psychostimulants might modify the relationship between reward value and social behaviors. These modifications depend on the length of drug use, dosage, and age of the first use, leading or not to drug addiction (Bardo et al., 2013; Clare et al., 2021).

Considering that the neural circuits underlying the regulation and expression of social behaviors are highly overlapped with those targeted by psychostimulants, the aim of the present work is to give a general overview regarding the effects of illicit and prescription psychostimulants upon social behaviors in both animals and humans.

## 2 Social behaviors and the reward circuitry

The VTA is a critical brain region involved in social interaction and reward processes. DA neurons from VTA that project to NAc participate in the coding of social and non-social stimuli and this function is modulated by several neurotransmitters and neuropeptides, such as glutamate, GABA, oxytocin (OXT) and vasopressin (AVP), among others.

For instance, when VTA glutamatergic projections to NAc are activated, they stimulate DA release in NAc, and place preference behavior is reinforced. However, these glutamatergic projections can reinforce this behavior independently of DA release (Zell et al., 2020). OXT alters the readiness of the brain to orient to social stimuli via the activation of DA neurons in VTA (Groppe et al., 2013). Moreover, when activation occurs at this level, social interaction generates rewarding experiences by promoting prosocial behaviors (Hung et al., 2017). For example, it has been shown that in male Syrian hamsters there is an increase in VTA neuronal activation during social stimuli. Specifically, the number of c-Fos immunoreactive cells in the VTA of Syrian hamsters after OXT release was higher in males who experienced direct social interaction compared with socially naïve males. This indicates that social experience enhances the response of this area to social stimuli (Hung et al., 2017). Another work done in Syrian hamster demonstrated that activation of the OXT receptor (OXTR), but not the vasopressin type V1a receptor (V1aR) in the VTA is crucial for the encoding of social interactions as reward (Song et al., 2016).

The VP is a GABAergic nucleus with a small population of glutamatergic projections (Soares-Cunha and Heinsbroek, 2023). This nucleus is part of a larger network of limbic brain areas that mediates the salience of several rewards (food, social affiliation, sex, and others) (Soares-Cunha and Heinsbroek, 2023). It also acts as a central point for inputs from the medial amygdala (MeA), VTA, and lateral hypothalamus (LH), among other structures, and the VP projects back to its input sources, including the NAc and VTA for reciprocal information exchange (Ahrens et al., 2018; Zhou et al., 2022).

Considering the multiple connections with areas related to core processes related to reward (motivation, arousal, motor control), VP is seen as a control center that regulates limbic signals and translates them into motor outcomes (Ottenheimer et al., 2020; Soares-Cunha and Heinsbroek, 2023). Regarding motivated behaviors and natural rewards, in a study using cell specific fiber photometry it was shown that inhibition of glutamatergic neurons in VP reduces the animals’ ability to detect salient stimuli, and those neurons got activated in response to several reward related stimuli (of social and non-social nature) (Wang et al., 2020).

Fiber photometry recordings revealed that VTA GABA projection neurons respond to food (reward). This response is relatively invariable (the outcome does not change as animals learn that a cue predicts reward availability) and the extent of this response is completely correlated with the size and deliciousness of the reward. Moreover, VP neurons respond to both reward consumption and seeking–associated motor activity. Additionally, when the VTA-to-VP GABA pathway is chemogenetically stimulated, an increased activity in a portion of VP neurons responsive to reward is observed. Interestingly, this effect was shown only in those neurons related to motivated motor activity but not in those related to reward consumption (Zhou et al., 2022). Additionally, optogenetic stimulation of these neurons enhances the performance of freely moving mice in a cued reward task and maintains a high incentive for reward during a progressive ratio schedule of reinforcement (Zhou et al., 2022).

NAc is a significant structure of the ventral striatum involved in the responses related to reward, motivated and goal-directed behaviors, and drugs of abuse. (Ambroggi et al., 2011; West and Carelli, 2016; Piantadosi et al., 2018). For example, in young animals there is an increase in neuronal activity (c-Fos immunoreactivity) after social play (a motivated young social behavior) (van Kerkhof et al., 2014). Furthermore, GABA antagonists can modify the length of the play (van Kerkhof et al., 2013). In adults, sexual consummatory behavior increases DA release in NAc in males (Sun et al., 2018; Sun et al., 2020). Moreover, a study showed DA release in both naïve and pair-bonded males in response to pups in prairie voles (Lei et al., 2017).

Altogether, these data identify the importance of DA, glutamate, and GABAergic neurons within the VP-VTA-NAc circuit in the salience, learning, and seeking of natural rewards. Furthermore, the neuronal response is in accordance with the salience magnitude of the reward, and this does not change during a cue learning protocol, reflecting the importance of VP-VTA-NAc communication on reward and motivation for both natural and artificial rewards.

The PFC has a complex mix of inhibitory interneurons and receives inputs from neuromodulators such as acetylcholine (ACh), DA, and OXT, being suggested as a top-down control system for decision-making, goal-directed behaviors and complex cognitive processing (Miller and Cohen, 2001; Hoover and Vertes, 2007; Anastasiades and Carter, 2021). Indeed, the PFC projects to several brain areas (NAc, Hipp and amygdala) that are known to influence sociability (Franklin et al., 2017). Lesions of regions within the PFC in rodents have demonstrated its importance in social functioning. For example, the lesion of the rat orbitofrontal cortex increases aggressive behavior (Rudebeck et al., 2007). Furthermore, it has been shown that, in rats, PFC was activated (c-Fos activity) during social interaction (Wall et al., 2012), also showing the involvement of the PFC in social behavior.

It has been proposed that the medial PFC (mPFC) is also pivotal for normal social behavior. A portion of mPFC neurons increases activity when social stimuli (unfamiliar mouse), but not when non-social stimuli (an object) are presented (Levy et al., 2019). In humans, different reports show impairments in mPFC activity and altered social behavior in individuals with autism spectrum disorder (von dem Hagen et al., 2013; Karen Pierce et al., 2004).

In summary, the PFC is important for generating appropriate social responses and controlling execution function, by evaluating and interpreting social information of the moment within the context of previous experiences (Franklin et al., 2017; Levy et al., 2019).

## 3 Psychostimulants and social behavior in animals

### 3.1 Social play

Social play is the first non-mother directed social behavior in young animals. It is characterized by exaggerated and distinct behavioral patterns related to social, sexual, and aggressive behavior (Vanderschuren et al., 2016). It is part of normal social behavior and is considered an indicator of emotional wellbeing, general health, and welfare of animals (Ahloy-Dallaire et al., 2018). This behavior is essential for social, cognitive, emotional, and motor abilities development, having a role in the environment and social context adaptation (Vanderschuren et al., 1997; Vanderschuren et al., 2016). Social play behavior is a highly pleasurable rewarding activity, especially in young individuals (Trezza et al., 2010) across species. This behavior starts around weaning and continues until early adolescence (Vanderschuren et al., 2016), where the type of response in the play depends on age and sex (Pellis and Pellis, 1987; Pellis and Pellis, 1990).

In rodents, social play is also known as ‘rough and tumble play or play fighting’ and it is characterized by highly physical and vigorous movement patterns between peers. In the context of juvenile rats, social play behavior can be divided into two basic components such as pouncing (interaction in which an animal nose rubs the partner’s nape “asking for play”) and pinning (interaction in which an animal stands over the ventral surface of another animal “wanting to play”), along with grooming, chasing, and boxing (Homberg et al., 2007; Argue and McCarthy, 2015a; Argue and McCarthy, 2015b). Furthermore, in rodents this type of behavior is accompanied by physical, facial, and vocal (ultrasonic vocalization) signals with ludic intention (Vanderschuren et al., 2016).

Thus, social play generates emotional excitement and bonding having a neurobiological function in development. Some studies have shown how animals with play deprivation generate higher levels of anxiety (Lukkes et al., 2009; Vanderschuren et al., 2016) and hence higher sensitivity to drugs (Whitaker et al., 2013; Lesscher et al., 2015; Heilig et al., 2016). Play is sensitive to several factors including motivational state, isolation periods and housing conditions. For example, in young Sprague Dawley rats, different housing conditions produce high or low levels of playing, and isolation usually diminishes general social interactions in young and adult rats regardless of sex (Varlinskaya and Spear, 2008).

Psychostimulants can profoundly alter social play behavior due to their effects on brain mechanisms involved in reward (Trezza et al., 2014; Knell, 2022). For instance, AMPH, MPH, and cocaine suppress social play. It has been shown that AMPH and MPH have a suppressive effect on social play through the stimulation of α2 adrenoceptors but not through DA receptors (Achterberg et al., 2014). The inhibitory effects of MPH on social play are mediated by a distributed network of prefrontal and limbic subcortical regions involved in cognitive control and emotional processes. Thus, microinjections of MPH in the infralimbic cortex, anterior cingulate cortex, basolateral amygdala and habenula produce a decrease in social play behavior (Achterberg et al., 2015). The motivation for social play is mediated by DA receptors stimulation and the suppression in the expression of play is mediated by stimulation of adrenergic α2 receptors, suggesting that the decrease in social play behavior promoted by MPH is regulated by a network that could work in parallel (Achterberg et al., 2016). Besides, it has been shown that chronic treatment with MOD in pre-adolescent rats, changes dopaminergic neurotransmission in the NAc decreasing DA release induced by a depolarizing stimulus, and suggesting a decrease in the ability of rats to perceive the rewarding effects of social play (Cid-Jofre et al., 2021).

In addition, cocaine, MPH and AMPH have demonstrated to impair social play behavior when are administrated acutely (Vanderschuren et al., 2008; Manduca et al., 2016), but not after chronic MPH (Bolanos et al., 2003). These data suggest that the extent of psychostimulant exposure is crucial for determining behavioral and molecular outputs in the brain reward circuit such as receptor expression, second messenger signaling, transporter function, and trafficking, among others (Anderson and Pierce, 2005).

### 3.2 Aggression

Aggressive behavior is a form of social communication, used to exert control over the social environment. It is characterized by a series of specific sequenced patterns that vary across the species in terms of frequency and duration. These are usually aimed at intruders considered rivals and are meant to suppress their reproductive success (Bartholow, 2018).

Aggressive behavior can be offensive or defensive. The offensive behavior is typically displayed by the resident and is characterized by introductory and threatening demonstrations towards the intruder intended to damage him, sometimes including an attack or behaviors such as aggressive groom, charge, and chase, among others (Koolhaas et al., 2013). The intruder usually displays in response a defensive behavior, characterized by the lack of initiative, evade, flee, jump, and others, which can result in non-intentional damage (Olivier and Young, 2002; Koolhaas et al., 2013).

It is important to note that aggression and violence are not the same. Aggressive behaviors are social responses considered normal and adaptive in animals and humans. On the other hand, violence is a harmful form of offensive aggression, given that it is a form of behavior regardless of the context that may include attacks towards vulnerable body spots such as paws, belly, and throat. In contrast, the aggression is usually directed to the back and the neck, both considered non-vulnerable targets for the attack (Koolhaas et al., 2013). It has been shown in mice a phenomenon known as “addiction to aggression.” Aggression is highly sought due to its gratifying effects, despite negative consequences in the short and long term, registering high rates of relapse, which reminds some features observed in drug addiction (Golden et al., 2019).

Several studies have focused on the impact of psychostimulants on aggressive behavior. Currently, mounting evidence shows that the use of drugs like AMPH, MAMPH, and ecstasy influences aggressive behavior, whereas the effect of MOD on this type of behavior has been less studied (Dawe et al., 2009; Machalova et al., 2012). Machalova and colleagues (Machalova et al., 2012) showed in an animal model of social conflict interaction (agonistic behavior model), that MOD inhibits attacks in aggressive mice and increases aggression in shy mice. On the other hand, acutely administrated MAMPH in aggressive mice produces a dose-dependent inhibition of aggressive behavior and an increase in timid behavior at high doses (Machalova et al., 2012). Finally, when they administered MDMA, both types of mice (aggressive and shy) showed an increase in the frequency of timid displays and a decrease in aggressive behavior was observed only in aggressive mice (Machalova et al., 2012). Similar results have been shown in BALB/cJ mice, an animal model of ADHD and conduct disorder, where the administration of 3 mg/kg intraperitoneal MPH produces a decrease in aggressive behavior while 10 mg/kg increases aggression, showing a dose-dependent effect (Jager et al., 2019). The first studies done with AMPH showed similar results (Miczek, 1974; Miczek and Tidey, 1989). Currently, the National Institute on Drug Abuse (NIDA, USA) directly relates the chronic use of MAMPH with violent behavior (NIDA, 2023). However, an increase in aggression levels due to the therapeutic use of AMPH has not been reported (O’Malley et al., 2022).

Regarding cocaine effects on aggression, controversial results have been reported. For instance, exposure to a single high dose of cocaine in adult rats produces a decrease in defensive behaviors in the resident-intruder paradigm (Alves et al., 2014), whereas when a social interaction test was used, the results were opposite (Rademacher et al., 2002). In isolated adult mice that receive cocaine in a single-binge administration, an increase in their defense behavior was observed (Estelles et al., 2004).

As indicated by the evidence, psychostimulants effects on aggressive behavior are diverse. This variability could be related to different paradigms, drug dose, age, type of administration (acute or chronic), and the specific neurobiological effects of each drug.

### 3.3 Maternal care

Maternal care is a complex behavior that directly influences the survival chances of the offspring (Fischer and O’Connell, 2018). This behavior includes building a nest and crouching over pups to keep them warm, nursing pups to provide nutrition, grooming them, protecting them from intruders, and retrieving pups back to the nest when they become displaced. The neurobiology of maternal care has been frequently studied in rodents, since females display very well characterized maternal behaviors towards their offspring that are easily distinguished and measured (Stolzenberg and Mayer, 2019).

Maternal care is sensitive to several environmental factors such as drugs of abuse, experience, housing conditions, isolation, and pharmacological manipulations. For instance, Perry and colleagues studied the effects of 3 days of MAMPH administration at three different doses on parental behavior in prairie voles. They found that the lowest dose (0.2 mg/kg) was enough to reduce the contact with the pups (Perry et al., 2019). When MAMPH injections (5 mg/kg) were used prenatally in pregnant rats, during the first or second half of the gestation period, several physiological and sensorimotor effects were observed in the offspring, although there were no differences in maternal behavior (Malinová-Ševčíková et al., 2014). On the other hand, repeated MPH (5 mg/kg) exposure during the lactation period impairs both maternal and adult offspring behavior. Regarding maternal behavior, the latency of retrieving pups was increased accompanied by a lower number of dams retrieving their pups (Ponchio et al., 2015).

In an interesting study with a fostering approach, the maternal behavior in juvenile offspring that were reared by dams exposed to intermittent cocaine was poor (Johns et al., 2007). Besides, chronic cocaine administration twice daily to pregnant rats for the entire gestational period elicited those dams to spend significantly less time next to the pups on *postpartum* day 1, and at *postpartum* day five, pups engaged in less ultrasonic vocalizations (Lippard et al., 2015). In contrast, another study showed that 10 days of cocaine administration to females prior to mating was enough to increase subsequent maternal behavior (Nephew and Febo, 2010). In particular, these mothers retrieved pups more quickly, spent more time taking care of the pups, and were more aggressive towards a male intruder on day 2 of lactation compared to control animals. However, no effects were observed on days 9 and 16 of lactation (Nephew and Febo, 2010).

In summary, maternal behavior is a set of complex behaviors towards pups with their survival as a major goal. As with other social behaviors, caring for pups is a rewarding experience that can be positively or negatively affected by psychostimulants.

### 3.4 Pair bonding and partner preference

Pair bonding is the social behavior associated with the ability to form intense and selective social attachments (pair bonds) usually, but not exclusively, related to sexual attraction and the partner preference for mating. The brain areas associated with this behavior are PFC, NAc and VP among others.

The rodent prairie vole (Microtus ochrogaster) is an animal widely used to study pair bonding and the effects of drugs of abuse on the neurobiological mechanisms behind this behavior. This species is highly social and monogamous (although now it is known that there are two species, one monogamous and the other promiscuous) and forms long-term and exclusive pair bonds with a partner after mating (McGraw and Young, 2010). In this case, preference for the partner over a stranger conspecific is considered a pair bond index and is usually measured as time or percentage of total time of close physical contact between the animals.

In adult prairie voles, it has been reported inhibition of partner preference after AMPH exposure in both sexes (Liu et al., 2010; Young et al., 2014). Notably, the effects of AMPH were observed exclusively on partner preference since other related behaviors such as mating frequency or locomotor activity were not affected. Studies performed in monogamous species have suggested that neuropeptides such as OXT and AVP facilitate pair bond (Rigney et al., 2022). For instance, it has been reported that OXT administration into the mPFC restores both partner preference and NAc DA levels, impaired by AMPH administration (Young et al., 2014). On the other hand, in agreement with the protective effects of social bonding on drug abuse, Liu and colleagues (Liu et al., 2011) have demonstrated that pair bonding prevents AMPH-induced CPP, through a D1R-mediated mechanism. Moreover, as shown in female prairie voles, AMPH exposure increases DA levels in the NAc but not in mPFC or VTA, increases D1R mRNA in the NAc and decreases D2R mRNA in VTA. These data indicate that the effects of AMPH upon pair bonding in female prairie voles exhibit nuclei and receptor specificity (Young et al., 2011).

In the same way, a study using a continuous 2 bottle choice procedure (one bottle with tap water and other bottle with MAMPH), showed that MAMPH had an inhibitory effect in partner preference, an effect that is accompanied by a decrease of OXT levels in the paraventricular nucleus of the hypothalamus (Hostetler et al., 2016).

Globally, these works highlight the profound effects of psychostimulants in pair bonding. Since, OXT modulates the DA system and the reward process, drugs acting on OXT receptors appear promising as treatments for conditions in which these behaviors are altered.

Table 1 summarizes the results of the studies evaluating the effects of different psychostimulants upon diverse social behaviors in animal models.

**TABLE 1:** Animal research summary in social play, aggression, maternal care, pair bonding and partner preference studies*.

Ref.	Drug	Dose	Treatment	Main results reported		Important factors considered
				Social activities	Neuro-chemical or –biological mechanism shown/suggested	
Social play behavior						
Achterberg et al. (2014)	AMPH	0.05–0.5 mg/kg s.c	Acute; 30min before test	0.2 and 0.5 mg/kg reduced play but not social exploration	Impacts negatively on social play through α-2 NE but not DA receptors	• Male Wistar rats PND26–28
	Cocaine	0.5–7.5 mg/kg s.c		5.0–7.5 mg/kg reduced play	Impacts negatively on social play through simultaneous increases in DA, NE, and 5-HT neurotransmission	• Experimental and partner rat received the same drug treatment
Achterberg et al. (2015)	MPH	5.0 µg/0.3 µL	Acute; 5min before test days (2 sessions of social play behavior test)	Infusions in ACC, IL, BLA and habenula reduced play without affecting social exploration or locomotor activity	Authors did not use pharmacological or cellular/molecular approaches for dissecting possible underlying mechanism	• Male Wistar rats PND26–27 underwent surgery
						• Experimental and partner rats received infusions
Cid-Jofre et al. (2021)	MOD	75 mg/kg i.p	Chronic; 14 consecutive days before test (from 22 to 35 PND)	Treatment reduced play and altered their structure without affecting social exploration	• Reverse dialysis of 70 mM K+ in NAc increased NAc extracellular DA in experimental animals to a lesser extent than control group	• Male Sprague Dawley rats PND 36
					• NAc D2R expression, DA and DOPAC content levels were similar in both groups	
Vanderschuren et al. (2008)	MPH	1.0 mg/kg s.c	Sub-chronic; 5 consecutive days	• 0.3–3.0 mg/kg dose dependently suppressed social play, but social exploration was not affected	Reduction in social play was mediated by α-2 NE receptor	• Male Wistar rats PND26–28
			Half of animals received 0.1 or 1.0 mg/kg, s.c. 30min before test	• 0.01 and 0.1 mg/kg did not alter pinning		
				• 1 mg/kg reduced social play as a whole		
				• 0.3 and 3.0 mg/kg suppressed pinning		
Manduca et al. (2016)	AMPH	0.03–1.0 µg/0.3 µL	Acute; 15min before test	• 0.03 and 0.1 µg/0.3 µL AMPH infusions increased play	• Direct stimulation of NAc DA receptors, either by AMPH-induced DA release or by apomorphine is necessary to increase social play in young male rats	• Male Wistar rats PND28 underwent surgery = NAc
					• Inhibiting DA uptake is not sufficient	• Experimental and partner rat received infusions
Bolanos et al. (2003)	MPH	2.0 mg/kg i.p	Chronic; 14 consecutive days, twice daily (PND 20 to PND 35)	• MPH chronic treatment did not affect play in male rats	Authors did not use pharmacological or cellular/molecular approaches for dissecting possible underlying mechanism	• Male Sprague Dawley rats
						• Social play test was carried out in PND40 (5 days after the last MPH injection)
Aggressive behavior						
Machalova et al. (2012)	MOD	2, 10, or 50 mg/kg i.p	30 min prior to test	• 2 and 10 mg/kg = decreased timid behavioral activities in timid mice	Authors did not use pharmacological or cellular/molecular approaches for dissecting possible underlying mechanism	• Male mice of the SPF ICR outbred strain
				• 50 mg/kg = did not change aggressive behaviors		
	MAMPH	1, 5, or 10 mg/kg i.p	15 min prior to test	• 1 mg/kg = reduced aggressive behavior in aggressive mice		• Individually housed mice were divided into 2 groups based on their behavior in the control interaction: a) aggressive or b) timid
				• 5 and 10 mg/kg = decreased aggressive and timid behavioral activities in aggressive mice		
				• All doses decreased sociable behavior activities in timid mice		
	MDMA	2.5,10, or 30 mg/kg i.p	30 min prior to test	• 2.5 mg/kg = in timid mice decreased sociable activities		
				• 10 and 30 mg/kg = . In timid mice decreased sociable activities and increased timid behavior		
				• All doses decreased aggressive behaviors and increase timid behaviors in aggressive mice		
Jager et al. (2019)	MPH	3 or 10 mg/kg i.p	20 min prior to test	• 3 mg/kg = prolonged attack latency and prevented escalation of aggression. Decreased social interaction	Authors did not use pharmacological or cellular/molecular approaches for dissecting possible underlying mechanism	• BALB/cJ mice
				• 10 mg/kg = increased number of bites and attacks		
Miczek (1974)	AMPH	0.05, 0.1, 0.5, 1.0 mg/kg i.m.	30 min prior to test	• Lower doses increase aggressive behaviors and higher doses decreased them	Authors did not use pharmacological or cellular/molecular approaches for dissecting possible underlying mechanism	• Sprague Dawley rats
Maternal/Paternal care behavior						
Perry et al. (2019)	MAMPH	0, 0.2 or 2.0 mg/kg, i.p	daily injections for 3 consecutive days	• 0.2 mg/kg = decreased maternal and paternal care	Authors did not use pharmacological or cellular/molecular approaches for dissecting possible underlying mechanism	• Adult male and female prairie vole PND60-122
				• 2.0 mg/kg = decreased paternal care		
Malinová-Ševčíková et al. (2014)		5 mg/kg s.c	ED1-11 or ED12-22	• There were no differences in any of the maternal activities between treated and control dams in both periods of injection		• Adult albino Wistar rats
Ponchio et al. (2015)	MPH	5 mg/kg p.o	LD 2–7	• Decreased maternal behavior	Levels of 5-HT were reduced in the MPH group compared to control	• Female Balb-c mice
Johns et al. (2007)	Cocaine	CC = GD1–20	Cocaine = chronic (CC), intermittent (IC)	• PND28 = maternal care was decreased in IC-treated dams	Authors did not use pharmacological or cellular/molecular approaches for dissecting possible underlying mechanism	• Sprague–Dawley rats PND28 (males and females) and PND60 (males only)
		IC = GD2, 3, 8, 9, 14, 15, 20 (and during *postpartum*)	Saline = chronic (CS), intermittent (IS)	• PND60 = maternal care was decreased in CC-treated dams		• Cross-fostering protocol = after parturition litters were either returned to their mothers or fostered to dams from a different group
		15 mg/kg s.c	Untreated (UN)			
Lippard et al. (2015)		GD 1–20	Cocaine = chronic (CC)	• PPD1 = CC dams spent more time on the UN and CS pup side than they spent on the CC pup side	• PPD 5 = CC dams had fewer OXT (+) cell bodies in the mPOA compared to UN dams	• Sprague–Dawley rats
		15 mg/kg s.c	Saline = chronic (CS)	• PPD5 = CC dams spent more time on the UN pup side than did UN and CS dams. Moreover, they touched/sniffed both UN and CC pup cages for a shorter duration than did UN and CS dams	• PPD 5 = CC dams showed a positive relationship between n° of OXT (+) cell bodies and preference-like behavior. Greater OXT expression was associated with higher engagement in touch/sniff behavior depending on the pups group	• Cross-fostering protocol = each test dam had two pup-providers (one UN and one CC) assigned to for postpartum testing
			Untreated = (UN)			
Nephew and Febo (2010)		15 mg/kg i.p	10-day treatment period plus at least 4 days of withdrawal	• LD 2 = increased maternal behavior	Authors did not use pharmacological or cellular/molecular approaches for dissecting possible underlying mechanism	• Long–Evans female rats
						• Saline and cocaine treatments ended 4 days prior to the mating period
						• Behavioral testing at LD 2, 9, 16
Pair bonding and partner preference						
Liu et al. (2010)	AMPH	1.0 or 5.0 mg/kg i.p	Once per day for 3 consecutive days	• 1.0 and 5.0 mg/kg = failed to show partner preferences, while not affecting mating frequency	• 1 mg/kg = increase in D1R, but not D2R, mRNA labeling within the NAc, compared to control males	• Male prairie vole about PND 90 when tested
					D1R blockade in the NAc eliminated the AMPH-induced impairment of partner preference formation	
Young et al. (2014)		0.2 or 0.5 mg/kg i.p		• 0.2 and 0.5 mg/kg = did not form partner preference, but instead form nonselective contact with the stimulus male during the partner preference test	• Lower OTR-ir levels in the mPFC than controls	Female prairie vole PND 90–120 when tested
					• Lower D2R-ir levels in the NAc than controls	
					• Higher [DA]ext in the NAc than controls	
					• OXT-PLC infusion restores mating and induced partner preference formation in treated subjects	
Hostetler et al. (2016)	MAMPH	100 mg/L in tap water	18 h/day for 3 consecutive days	• Treatment decreased partner preference	Fewer OXT (+) but not AVP(+) cells in the PVN of treated animals than controls	• Male and female prairie voles PND 60–120

*For methodological details see the original study.

AMPH, amphetamine; MPH, methylphenidate; MOD, modafinil; MAMPH, methamphetamine; MDMA, methylenedioximethamphetamine; DA, dopamine; NE, norepinephrine; 5-HT, serotonin; DOPAC, 3,4-Dihydroxyphenylacetic acid; ED, embryonic day; LD, lactation day; GD, gestation day; s.c. = subcutaneously; i.p. = intraperitoneal*;* p.o. = pers ors; i.m. = intramuscular*;* PPD, *postpartum* day; PND, post-natal day; ACC, anterior cingulate cortex; IL, infralimbic cortex; mPFC, medial prefrontal cortex; mPOA , medial preoptic area; NAc, nucleus accumbens; BLA, basolateral amygdala; PVN, paraventricular nucleus; OXT, oxytocin; AVP, vasopressin.

## 4 Psychostimulants and social behaviors in humans

Psychostimulants could also affect human social skills and behavior (Henry et al., 2009; Fox et al., 2011; Morgan and Marshall, 2013; Preller et al., 2014; Shreffler et al., 2022). However, as seen with animal models, the effects of psychostimulants upon social behavior can be controversial and even opposite depending on the type of drug analyzed, whether they are used in clinical or recreational/abuse contexts, the time of use, the age of the user, etc. Thus, for example, it has been shown that in general terms, chronic psychostimulant (e.g., cocaine or MAMPH) abusers tend to have some impairment in key social skills (Henry et al., 2009; Fox et al., 2011; Morgan and Marshall, 2013; Preller et al., 2014; Shreffler et al., 2022) and reduced social networks, and also show a series of alterations in brain networks underlying social behaviors (see, for example, (Aron and Paulus, 2007; Monterosso et al., 2007; Kim et al., 2010; Mackey and Paulus, 2013)).In contrast, patients using psychostimulants in clinical settings (e.g., MPH for the treatment of ADHD) exhibit improvements in some of the same aforementioned social skills (Fantozzi et al., 2021a; Fantozzi et al., 2021b; Levi-Shachar et al., 2021). Beyond these global considerations, most of the reports highlight that there is a lack of research in humans regarding psychostimulants and social behaviors since the majority of the works are related to their physiological effects, addictive properties, and potential treatments for their abuse. Here, we give an overview of some recent studies that show a relationship between psychostimulant consumption and modifications in sociality, empathy and aggression in humans.

### 4.1 Pro sociality and empathy

One of the most studied drugs in relation with social behavior is MDMA. In 2012, MDMA was estimated to be the 3rd recreational drug among adults (between 18 and 25 years old) and in the same year, nearly 869,000 consumed it for the first time in the USA (Substance Abuse and Mental Health Services Administration, 2014). In addition, MDMA is under examination as a potential counterpart for psychotherapy (Feduccia et al., 2023; Kangaslampi and Zijlmans, 2023). In various reports it has been described that MDMA has a prosocial effect. For instance, Wardle and de Wit (Wardle and de Wit, 2014) analyzed the effects of MDMA using a three-session within-subjects design over 36 healthy volunteers of both sexes. In females only, MDMA (1.5 mg/kg) both increased smiling (zygomatic muscle activity) and reduced frown (corrugator muscle activity) in response to happy facial expressions, indicating more positive reactions to positive facial expressions. Therefore, MDMA altered emotional perception processing and response. It also impacts behavior and perception in an actual social scenario with a partner (Wardle and de Wit, 2014).

In a non-social and controlled laboratory setup, the administration of MDMA (from 0.5 to 2.0 mg/kg) increased (relative to placebo) self-report ratings of a broad range of socially relevant mood states. In this line, there is some evidence suggesting that MDMA prosocial effect depends on the social setting used in research. When MDMA (1.0 mg/kg single administration) was analyzed in volunteers, the increased feelings of confidence only appeared in subjects that were accompanied by other participants (exposed to MDMA too) (Kirkpatrick and de Wit, 2015). Interestingly, MDMA (0.75 mg/kg) increased the rating of loneliness (Bedi et al., 2010) in individuals who received the drug alone in isolated laboratory setups. In a study of free speech, MDMA increased the use of social and sexual words, as well as words referring to death, (Baggott et al., 2015), suggesting that consequences on speech reflect underlying alterations in social mood states. Thus, these findings suggest that MDMA generates prosocial feelings and different mental states in humans in controlled laboratory conditions that depend on social settings.

Studying several domains of social cognition in cocaine, MAMPH, and non-medical MPH users, interesting results have been described. For instance, a negative impact in the total performance for emotion cognition was observed only in MAMPH users. For emotional empathy, cocaine and MAMPH score lower than controls. Regarding cocaine users they show decreased social and moral decision-making, and lower social reward compared to controls [reviewed in (Quednow, 2017). As we mentioned previously, social network size is influenced by psychostimulant abuse. In both cases, cocaine and MPH users exhibit smaller social networks (Quednow, 2017).

More recently, social decision-making was analyzed in males with MAMPH use disorder. Applying a modified dictator monetary game where two scenarios were present (disadvantageous and advantageous), the pro-sociality behavior was measured. If the subject chose no money or less money for themselves against the hypothetical strangers, the choice was selected as pro-social. In the disadvantageous context, males with MAMPH use disorder made fewer pro-social choices compared to controls, however, in the advantageous context authors reported no differences in pro-social choices between groups (Li et al., 2022). Although the pro-social decisions could be different if the interaction were face to face, this study suggests that in disadvantageous scenarios MAMPH male users have less consideration of other’s benefits as compared to healthy controls.

On the other hand, there is evidence that ADHD patients have some problems with emotion recognition and social skills. MPH is the first-line medication for this disorder and several works have studied the effect of the drug on different aspects of social performance. Alkalay and Dan (Alkalay and Dan, 2022), after reviewing 15 studies on the impact of MPH on social performance in children between 6 and 14 years old with ADHD, concluded that MPH seems to improve the social deficit after weeks of treatment, increasing the capacity to recognize emotions and decreasing conflictive behaviors. However, as mentioned, these authors recognize that still controversial results exist and further studies are required to confirm conclusions.

On the other hand, the administration of lisdexamphetamine (LDX) to parents diagnosed with ADHD improved parenting behaviors. The pharmacological intervention reduced ADHD symptoms -negative talk during free play and quiet time components. Also, parents treated with LDX made over four times more praising statements, reduced their number of verbalizations during the homework task, and reduced the percentage of demands during tasks (Waxmonsky et al., 2014).

### 4.2 Aggressive behavior

High aggression is a problematic social behavior that can be explained, among other causes, by a deficit of social cognition skills that could be impaired by the use of drugs. Impulsivity, a characteristic related to drug abuse, has also been associated with aggressive behaviors. In addition, although not well studied, the use of psychostimulants seems to be a cause of aggression. A study performed in medical students assessed the relationship between the use of psychostimulants for non-medical reasons and aggressive-hostility behavior. The study was carried out using a 73-question anonymous survey that included the Zuckerman–Kuhlman Personality Questionnaire in which aggressive behavior is described as a “tendency to express verbal aggression and show rudeness” among other qualities such as “thoughtlessness, vengefulness, spitefulness, quick temper and impatient behavior.” Results showed that the use of psychostimulants is related to aggressive behavior at least in this student sample (Bucher et al., 2013).

Among psychostimulants, several studies have shown that the use of amphetamine-related drugs, in particular MAMPH, increases aggressive behaviors or trait aggression (Sekine et al., 2006; Plüddemann et al., 2010; Payer et al., 2012; Lederer et al., 2016). A study assessed the magnitude of deficit in social cognition skills and its relation with aggression in MAMPH dependent individuals, compared with MAMPH users with psychosis and a control group. The criteria used were the facial morphing “emotion recognition task” with emotions such as anger, fear, happiness, and sadness, the “reading the mind in the eyes task” and the aggression questionnaire. The MAMPH dependent groups showed impairment in social cognition skills and higher levels of aggression, although there were no associations between them (Uhlmann et al., 2018).

Although it is well known that the use of MAMPH increases aggression, little is known about the underlying biological mechanisms. It has been shown that MAMPH withdrawal increases aggressive levels and decreases the expression of serotonin transporters in several brain regions like the midbrain, striatum and cortex (Sekine et al., 2006). Other studies have shown that MAMPH dependent individuals exhibiting higher aggression levels also showed higher activity in PFC and occipital cortex (Payer et al., 2012) and a decrease of frontal white matter (Lederer et al., 2016). Altogether these results suggest that MAMPH abuse increases aggression due to, at least in part, the alteration in frontal cortex functions.

The use of alcohol is related to aggression, and on many occasions people combine the use of alcohol with other drugs. It has been shown that in amphetamine-type stimulant users, the combination with alcohol increases aggression and hostility only in MAMPH users but not in those who combine alcohol with MDMA (EllenLeslie et al., 2017). Another risk factor that could increase aggressive behavior in MAMPH users is post-traumatic stress disorder (Wahlstrom et al., 2015). These results highlight the importance of different risk factors that could increase the levels of aggression in psychostimulant users.

Regarding cocaine, less is known about the relationship between this drug and aggression. In a 2002 study, Moeller and colleagues (Moeller et al., 2002) investigated the effects of cocaine on impulsivity. In brief, impulsivity was measured in both cocaine dependent subjects and controls using an impulsiveness scale and a monetary delayed behavioral task using a computer. The results showed a higher impulsivity in cocaine dependent volunteers compared to control individuals and this outcome was not related to a history of aggressive behavior (Moeller et al., 2002). Importantly, the impulsiveness scale applied did not distinguish between social and non-social related impulsive behavior. In another study, detoxified cocaine-dependent patients had higher levels of physical aggression and hostility, and also, higher levels of dysfunctional impulsivity than healthy control subjects (Roozen et al., 2011). On the other hand, in the context of ADHD treatment, it has been shown that the treatment with MPH was effective on aggressive behavior when ADHD is comorbid with oppositional defiant disorder and aggression (Masi et al., 2017). However, this study has several limitations (non-randomized, non-blinded and few patients), and therefore more studies are needed to elucidate the effect of psychostimulants on aggressive behaviors, when used as a therapeutic treatment.

Table 2 summarizes the results of the studies evaluating the effects of different psychostimulants upon diverse social behaviors in humans.

**TABLE 2:** Human research summary in social behaviors.

Ref.	Drug	Dose	Treatment	Main results reported	Important factors considered
				Social activity	
Pro sociality and empathy					
Wardle and de Wit (2014)	MDMA	0.75 and 1.5 mg/kg	Three-sessions treatment; each session separated by a minimum of 7 days	Increased smiling and reduced frown, altering emotional processing and response. Increased use of positive emotion words	36 healthy volunteers, ages 18–35, with previous ecstasy use. Drug administrated in isolated laboratory setups
				1.5 mg/kg increased ratings of perceived regard during the social interaction and increased ratings of empathy	
Bedi et al. (2010), Kirkpatrick and de Wit (2015)		1.5 and 1 mg/kg	Acute treatment. Measures evaluated 2–4 min after drug administration	Increased feelings of confidence when others accompanied subjects. Also increased ratings of attractiveness of the other person and increased social interaction	Healthy Young adult volunteers aged 18–35 with moderate MDMA experience (4–80 lifetime uses)
Quednow, 2017; Li et al. (2022)	MAMPH	Chronic use		Lower emotional empathy, smaller social networks and increased non pro social choices	Non-medical use in males and females with drug use disorder
Parenting behavior					
Waxmonsky et al. (2014)	LDX	30, 50 or 70 mg/day	3-week open label titration to determine optimal dose for each. Evaluation begun the week after dose optimization	Improved parenting behaviors, reduced negative talk during the free play and quiet time components. Also, increased praising statements, reduced verbalizations during homework task and the percentage of demands during tasks	Parents with ADHD diagnostic (27% male)
Aggressive behavior					
Sekine et al. (2006), Plüddemann et al. (2010), Payer et al. (2012), Lederer et al. (2016)	MAMPH	Chronic use		Increased aggressive behaviors, violence, or trait aggression. Higher levels of physical aggression and anger	1561 humans (53% females) between 12–17 years with MAMPH use disorder
Masi et al. (2017)	MPH	5–10 mg/day	Monotherapy during the follow up. Titrations of 5–10 mg no more frequently than a 5 days intervals	Reduced aggressive behavior	20 males between 6 and 12 years with diagnosed ADHD and oppositional defiant disorder and aggression; without previous pharmacotherapy

## 5 Concluding remarks and future considerations

There is mounting evidence, both from animal models and human studies, showing that psychostimulants can significantly affect social behaviors. This is not surprising considering that the neural circuits underlying the regulation and expression of social behaviors are highly overlapped with those targeted by psychostimulants, which in most cases have strong rewarding and consequently addictive properties. Thus, it is also not surprising that the effects of psychostimulants on these behaviors can be very different depending on numerous variables such as the type of drug considered, acute *versus* long-term use, clinical *versus* recreational consumption or the presence or absence of concomitant risk factors (e.g., neuropsychiatric comorbidities, simultaneous consumption of other drugs, etc.), to name a few.

Furthermore, even though we used that terminology in this work, we think that considering psychostimulants as a relatively homogeneous drug class might be a misleading approach. Thus, the diverse effects on monoamines, i.e., more pronounced effects on DA and/or NE and/or 5-HT, as well as their differential pharmacodynamics regarding monoamines uptake (blockers, releasers, partial releasers, etc.) should be considered carefully when assessing the impact of different psychostimulants upon social behaviors.

In our opinion, psychostimulants-induced changes in social behaviors, either prosocial or antisocial, should be always monitored, particularly when using this type of drugs in therapeutic contexts. This assessment will be not only beneficial to establish some specific behavioral effects and how these can translate to neurochemistry, but also would shed light on how this dimension can be used/modified to improve the desired clinical outcomes and to reduce the risk of abuse and/or addiction. Indeed, studies in rodents have shown that the interaction with a partner during the acquisition of addictive behavior or relapse decreases the chances of developing addiction (noteworthy, opposite results were found if the partner also uses the drug) (Sampedro-Piquero et al., 2019; Venniro et al., 2022). Therefore, it is essential to consider the reward elicited by social interaction as an important factor that could improve addiction treatments.

Despite these considerations, it is clear that much more research is needed to have a better understanding of the physiological mechanisms underlying different social behaviors and how they are affected by diverse psychostimulants. This seems to be of particular relevance in a scenario of a growing consumption of this type of compounds.
